# Altered synaptic ultrastructure in the prefrontal cortex of Shank3-deficient rats

**DOI:** 10.1186/s13229-020-00393-8

**Published:** 2020-11-17

**Authors:** Sarah Jacot-Descombes, Neha U. Keshav, Dara L. Dickstein, Bridget Wicinski, William G. M. Janssen, Liam L. Hiester, Edward K. Sarfo, Tahia Warda, Matthew M. Fam, Hala Harony-Nicolas, Joseph D. Buxbaum, Patrick R. Hof, Merina Varghese

**Affiliations:** 1 Nash Family Department of Neuroscience, Hess Center for Science and Medicine, Icahn School of Medicine at Mount Sinai, 1470 Madison Avenue, New York, NY 10029 USA; 2 Friedman Brain Institute, Icahn School of Medicine at Mount Sinai, New York, NY USA; 3 Seaver Autism Center for Research and Treatment, Icahn School of Medicine at Mount Sinai, New York, NY USA; 4 Department of Psychiatry, Icahn School of Medicine at Mount Sinai, New York, NY USA; 5 Unit of Psychiatry, Department of Children and Teenagers, University Hospital and School of Medicine, Geneva, Switzerland; 6 Department of Legal Medicine, University Hospital and School of Medicine, Geneva, Switzerland; 7 Department of Pathology, F. Edward Hébert School of Medicine, Uniformed Services University of the Health Sciences (USU), Bethesda, MD USA; 8 Present Address: Psychology Department, Rutgers University Brain Imaging Center (RUBIC), Rutgers University, Newark, NJ 07102 USA

**Keywords:** Autism spectrum disorder, Phelan–McDermid syndrome, Synapse morphology, Electron microscopy

## Abstract

**Background:**

Deletion or mutations of *SHANK3* lead to Phelan–McDermid syndrome and monogenic forms of autism spectrum disorder (ASD). *SHANK3* encodes its eponymous scaffolding protein at excitatory glutamatergic synapses. Altered morphology of dendrites and spines in the hippocampus, cerebellum, and striatum have been associated with behavioral impairments in Shank3-deficient animal models. Given the attentional deficit in these animals, our study explored whether deficiency of *Shank3* in a rat model alters neuron morphology and synaptic ultrastructure in the medial prefrontal cortex (mPFC).

**Methods:**

We assessed dendrite and spine morphology and spine density in mPFC layer III neurons in *Shank3*-homozygous knockout (*Shank3*-KO), heterozygous (*Shank3*-Het), and wild-type (WT) rats. We used electron microscopy to determine the density of asymmetric synapses in mPFC layer III excitatory neurons in these rats. We measured postsynaptic density (PSD) length, PSD area, and head diameter (HD) of spines at these synapses.

**Results:**

Basal dendritic morphology was similar among the three genotypes. Spine density and morphology were comparable, but more thin and mushroom spines had larger head volumes in *Shank3*-Het compared to WT and *Shank3*-KO. All three groups had comparable synapse density and PSD length. Spine HD of total and non-perforated synapses in *Shank3*-Het rats, but not *Shank3*-KO rats, was significantly larger than in WT rats. The total and non-perforated PSD area was significantly larger in *Shank3*-Het rats compared to *Shank3*-KO rats. These findings represent preliminary evidence for synaptic ultrastructural alterations in the mPFC of rats that lack one copy of *Shank3* and mimic the heterozygous loss of *SHANK3* in Phelan–McDermid syndrome.

**Limitations:**

The *Shank3* deletion in the rat model we used does not affect all isoforms of the protein and would only model the effect of mutations resulting in loss of the N-terminus of the protein. Given the higher prevalence of ASD in males, the ultrastructural study focused only on synaptic structure in male *Shank3*-deficient rats.

**Conclusions:**

We observed increased HD and PSD area in *Shank3*-Het rats. These observations suggest the occurrence of altered synaptic ultrastructure in this animal model, further pointing to a key role of defective expression of the Shank3 protein in ASD and Phelan–McDermid syndrome.

## Background

Autism spectrum disorder (ASD) is a neurodevelopmental disorder affecting 1 in 59 children in the USA [1]. Individuals diagnosed with ASD show difficulty in social interaction and communication and restrictive, repetitive behaviors from an early age [2]. ASD is a common comorbid condition in individuals with Fragile X syndrome, Rett syndrome, Phelan–McDermid syndrome (PMS), and other genetic disorders [3]. Studying the cellular mechanisms affected by the genes identified in these monogenic disorders has helped to understand the pathophysiology underlying ASD better (reviewed in [4]).

Deletions in chromosome 22q13, which encompasses several genes including *SHANK3*, cause PMS (also known as deletion 22q13.3 syndrome) [5, 6]. Deletion and point mutations of *SHANK3* also cause PMS [7–9], and haploinsufficiency of *SHANK3* is linked to many of the neurological symptoms in subjects with PMS [7, 9–12]. Children with PMS show global developmental delay, delayed or absent speech, moderate-to-severe intellectual disability, hypotonia, seizures, and psychiatric features including ASD, attention-deficit/hyperactivity disorder (ADHD), and bipolar disorder [6, 7, 13–16]. Mutations of *SHANK3* have also been identified as a major monogenic cause of ASD [17, 18].

*SHANK3* encodes the SH3 and multiple ankyrin repeat domains protein 3 that belongs to a family of proteins also including Shank1 and Shank2. The Shank proteins interact with multiple other proteins, including cell adhesion proteins at the synapse, cytoskeletal proteins within the dendritic spine, and, via other scaffolding proteins, with ionotropic and metabotropic glutamate receptors (mGluRs) at the postsynaptic density (PSD) of excitatory synapses [19–22]. The Shank proteins share five protein–protein interaction domains: from the N-terminus to the C-terminus, these are a multiple ankyrin repeats domain (ANK), an SRC homology domain 3 (SH3), a PSD-95/Disc Large homolog-1/Zonula occludens-1 domain (PDZ), a proline-rich cluster domain (PRC), and a sterile alpha-motif domain (SAM) [23]. All three Shank family members share domain structures that are 60–90% similar except for the PRC domain (33–40%) [21]. Guanylate kinase-associated protein (GKAP) bound at the PDZ domain and Homer bound at the PRC domain of Shank proteins enable recruitment of ionotropic *N*-methyl-d-aspartate (NMDA) receptors and mGluRs, respectively, to the PSD [20, 24]. Shank3 also directly interacts with subunits of ionotropic amino-3-hydroxy-5-methylisoxazole-4-propionic acid (AMPA) receptors at its PDZ domain [25, 26]. Shank proteins interact with PSD scaffolding proteins and cytoskeletal actin, mediated through cortactin binding at their PRC domains, to regulate the structure of dendritic spines [27]. Thus, all three Shank proteins have roles in assembling and stabilizing PSD complexes and in regulating spine structure, driven by activity at the synapse [20, 28, 29], and their altered expression may contribute to the behavioral deficits in ASD and PMS.

Individuals with ASD have deficits in social communication and restricted, repetitive, or unusual sensory-motor behaviors. A possible reason for the deficits in social communication and interaction shown by subjects with ASD is their difficulty understanding the emotional behavior of others [1, 2]. The prefrontal cortex (PFC) is known for its role in cognitive control, coordinating memory, planning, and executive activity [30, 31]. The orbitofrontal cortex in the PFC is also a major part of the network that processes empathy and social behavior [32]. Structural and functional abnormalities in the PFC have been demonstrated in patients affected by ASD [33–38]. As 80% of PMS patients present with ASD, the PFC could be similarly altered in PMS. Other psychiatric features seen in PMS [6, 13], including attentional deficit [39, 40], anxiety [41], bipolar disorder [42], and schizophrenia [43, 44], as well as comorbidities in ASD, such as ADHD, intellectual disability, anxiety, irritability and aggression, epilepsy, and sleep disturbances [45, 46], also implicate the PFC.

Here, we use a Shank3-deficient rat model to investigate potential dendritic and synaptic changes in the PFC caused by mutations in *Shank3*. The infralimbic and prelimbic cortex in the rat are generally considered to be homologous in function to the medial PFC (mPFC) in humans [47], as these regions in the rat are required for working memory tasks, attentional tasks, emotional regulation, behavioral flexibility, and forms of associative learning [48–52]. Given the prominent role of Shank3 at the PSD [53, 54] as well as the synaptic plasticity deficits in the mPFC and the attentional deficit seen in this Shank3-deficient rat model [55], we hypothesized that loss of Shank3 would affect dendrite arborization, spine density and morphology, synapse density, and ultrastructure of dendritic spines in the PFC of Shank3-deficient rats.

## Methods

### Animals

We used the *Shank3*-deficient rat model created by zinc-finger nuclease technology. This rat model harbors a deletion of 68 base pairs in exon 6 of the ANK domain, generating a stop codon that truncates the expression of the full-length Shank3 protein [55]. A similar truncation was observed in a patient with PMS who carried a c.601–1G > A mutation in *SHANK3* [56]. The resulting reduction of Shank3 expression in rats causes deficits in social recognition memory and attention [55]. For iontophoretic dye injections and 3D reconstruction of dendrite and spine morphology, we used both male and female rats (*n* = 18) at 5 weeks of age, with equal number of rats of each sex and 6 rats (3 males and 3 females) of each genotype. For electron microscopy, we used male rats (*n* = 18) at 5 weeks of age from three groups (*n* = 6 per genotype): wild-type (WT), *Shank3*-heterozygous (*Shank3*-Het) and *Shank3*-homozygous knockout (*Shank3*-KO). Our study followed the National Institute of Health Guidelines for the Care and Use of Experimental Animals, and all animal protocols were approved by the Institutional Animal Care and Use Committee at the Icahn School of Medicine at Mount Sinai.

### Perfusion and tissue processing

Rats were anesthetized using 30% chloral hydrate and intracardially perfused with 1% paraformaldehyde (PFA) in phosphate buffer (PB, pH 7.4) for 2 min, followed by a fixative comprising 4% PFA and 0.125% glutaraldehyde in PB for 10 min. The brains were immediately dissected, post-fixed overnight at 4 °C in 4% PFA and 0.125% glutaraldehyde in PB, and then transferred to phosphate-buffered saline (PBS) with 0.1% sodium azide at 4 °C until sectioned. The brains were hemisected, and the right hemisphere was cut into 250-µm-thick sections using a vibratome (VT1000S, Leica Microsystems, Buffalo Grove, IL). For electron microscopy samples, the sections were cryoprotected in a graded glycerol/PB solution after which the mPFC was microdissected into blocks that underwent cryosubstitution and low-temperature embedding as described earlier [57]. Five consecutive ultrathin sections were cut at 90 nm using a diamond knife (Diatome, Hatfield, PA) on an ultramicrotome (Reichert-Jung, Depew, NY) and mounted onto a Formvar-supported slot grid (Electron Microscopy Sciences, Hatfield, PA).

### Cell loading and confocal microscopy

#### Intracellular dye injection

Coronal sections of 250-µm thickness were incubated in 250 ng/ml 4′, 6-diamidino-2-phenylindole (DAPI, Sigma-Aldrich, St. Louis, MO) for 5 min to enable identification of the mPFC cortical layers. The sections were mounted on nitrocellulose membrane filters, immersed in ice-cold PB, and neurons in layer III of the mPFC were iontophoretically injected with 5% Lucifer Yellow (Invitrogen, Carlsbad, CA) in distilled water under a direct current of 3–8 nA until the dye filled the distal ends of the dendrites. Neurons selected for injection were spaced so as to avoid overlapping of their dendrites. Six to eleven neurons were injected per section. The sections were mounted with Fluoromount-G (Southern Biotech, Birmingham, AL) on gelatin-coated glass slides.

#### Reconstruction of basal dendrites

Pyramidal neurons were included for reconstruction if they were within layer III of the PFC, were completely filled, and showed intact tertiary branches within at least 50 µm of the soma. Such neurons were reconstructed in 3 dimensions under a 40×/1.3 N.A. Plan-Neofluar oil immersion objective on a Zeiss Axiophot microscope equipped with a motorized stage (Ludl Electronic Products, Hawthorne, NY), a Microfire video camera (Optronics, Tulsa, OK), and Neurolucida morphometry software (v. 11.11.3, MBF Bioscience, Williston, VT). Using Neurolucida Explorer software (MBF Bioscience), total dendritic length and number of intersections per 30-µm incremental radial distance from the soma were analyzed by the Sholl method [58].

#### Immunofluorescence and confocal microscopy of dendritic spines

After reconstruction of dendrites, the sections with Lucifer Yellow-filled neurons were stained by immunofluorescence to amplify the signal for confocal imaging of dendritic spines. Briefly, the slides were de-coverslipped, sections were washed in PBS and incubated at 4 °C overnight in biotinylated rabbit anti-Lucifer Yellow (1:500 in PBS with 0.6% Triton X-100, A5751, ThermoFisher, Waltham, MA). After several washes in PBS, the sections were then incubated for 2 h at room temperature with streptavidin-Alexa Fluor 488 conjugate (1:500 in PBS with 0.6% Triton X-100, S32354, Invitrogen, Carlsbad, CA). The sections were washed and mounted with Fluoromount-G on gelatin-coated glass slides and dried for at least 24 h prior to imaging. To select dendritic segments, whole cells were imaged on a CLSM 780 upright microscope (Carl Zeiss Microscopy, Jena, Germany) using a 20×/0.8 M27 Plan-Apochromat objective and a Ar/Kr laser at an excitation wavelength of 488 nm. Confocal stacks were acquired at 512 × 512 pixel resolution with a Z-step of 1 µm, a pinhole setting of 1 Airy Unit, and optimal settings for gain and offset. Basal dendritic segments at 50 µm from the soma were selected for high-resolution imaging if they met the following criteria: not a primary dendrite, no overlap with other dendrites or branching that would obscure spines, not too deep in the section, and parallel or at acute angles to the coronal plane. Dendritic segments (average length = 25 µm) were imaged using a 100x/1.46 Oil DIC Plan-Apochromat objective, and stacks were acquired at 512 × 512 pixel resolution with a Z-step of 0.1 µm, optical zoom of 3.3×, a pinhole setting of 1 Airy Unit, and optimal settings for gain and offset. The stacks were imaged with approximately 1 µm above and below the segment so as to fully include all spines. Three z-stacks were imaged from each neuron. Confocal stacks were deconvolved using an iterative blind deconvolution algorithm (AutoQuant X version X3.0.1, MediaCybernetics, Bethesda, MD).

#### Spine reconstruction and analysis

The deconvolved stacks were analyzed using Neurolucida 360 (version 2019.2.1; MBF Bioscience) to determine spine density and morphology. Neurolucida 360 allows for semiautomated reconstruction of dendrites and spines and classification of spines. Spines with their head diameter (HD):neck diameter ratio less than 1.1 were classified as stubby. Thin spines had an HD:neck diameter ratio greater than 1.1 and maximum HD less than 0.35 µm. Mushroom spines had an HD:neck ratio diameter greater than 1.1 and maximum HD greater than 0.35 µm. Filopodia had length greater than 3 µm. Six rats per genotype, 6 neurons per rat, and 3 dendrites per neuron were analyzed and manually checked by a trained investigator blind to the groups, with each segment manually inspected and corrected by a second investigator. Data from the reconstructions were exported using Neurolucida Explorer (MBF Bioscience). Analysis was conducted in R, accompanied by the use of the packages readxl and writexl [59], where the data were aggregated by spine type and averaged across each cell and then each animal.

### Ultrastructural analyses

Serial sections from each animal were imaged at 75 kV on an H-7000 transmission electron microscope (Hitachi High Technologies America, Clarksburg, MD) with an AMT Advantage CCD camera (Advanced Microscopy Techniques, Woburn, MA). Fourteen to 17 sets of images were collected from layers II and III in the mPFC in the five serial sections at a magnification of 17,000×. Based on a previous volume estimate of the prelimbic layer III in rats at 0.6 mm^3^ [60], a total disector volume of 2.6 × 10^−7^ mm^3^ from 17 sampling sites per animal would represent a sampling fraction of approximately 0.00004%. Electron micrographs were adjusted for brightness, contrast, and sharpness, and morphological analysis was carried out using Photoshop CS5 (version 12.0, Adobe, San Jose, CA).

**Fig. 1:**
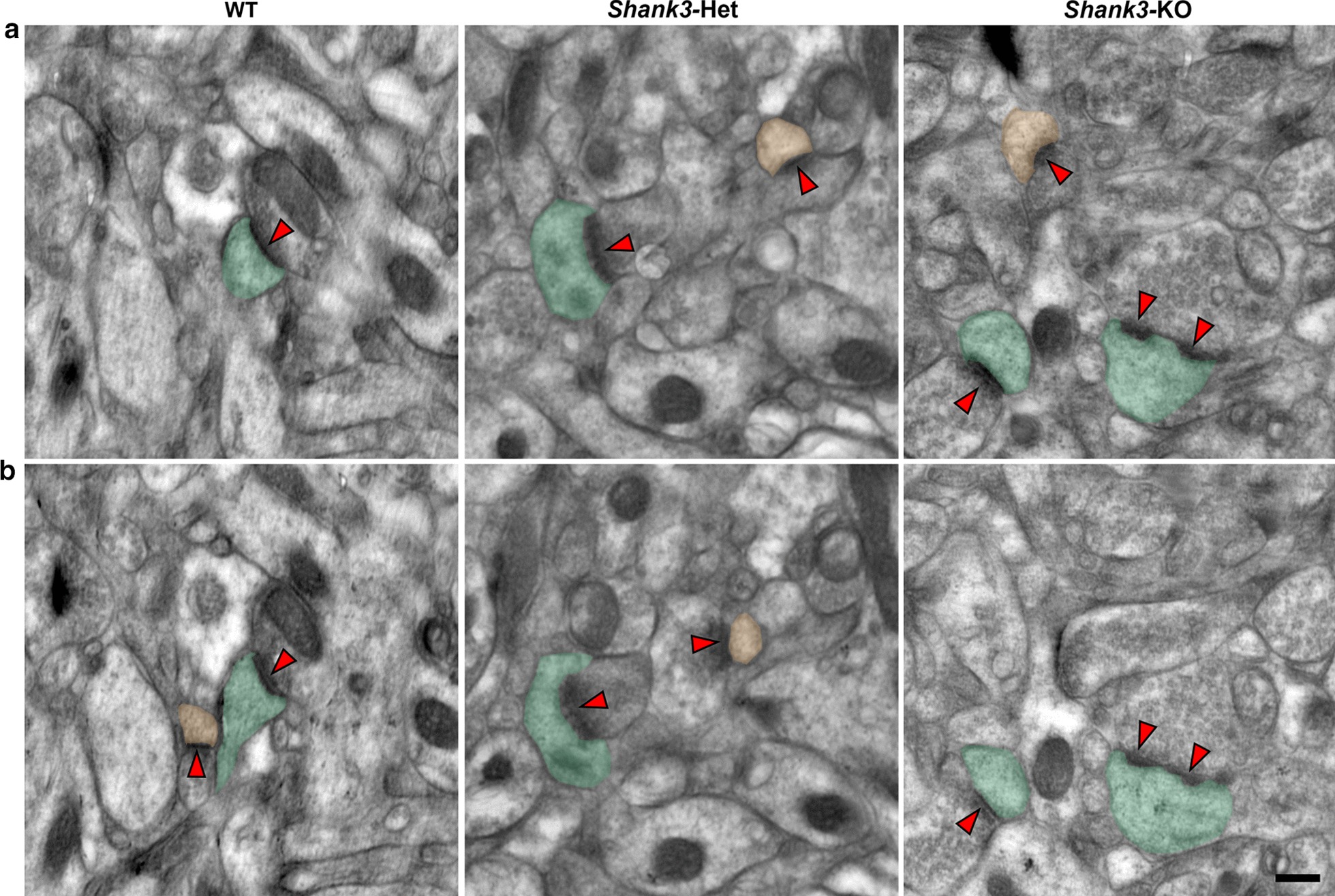
Disector method for estimation of synapse density. Electron micrographs showing examples of **a** reference layers with **b** their associated lookup layers. The orange masks indicate the postsynaptic spine of unique synapses, seen only in the reference layer or the lookup layer. The green masks show the postsynaptic spine of synapses seen in both layers. The red arrowheads indicate electron-dense PSDs. Note the split PSDs in the perforated synapses. Scale bar = 250 nm. WT, wild-type; *Shank3-*Het, *Shank3* heterozygotes; *Shank3-*KO, *Shank3* knockouts

**Fig. 2:**
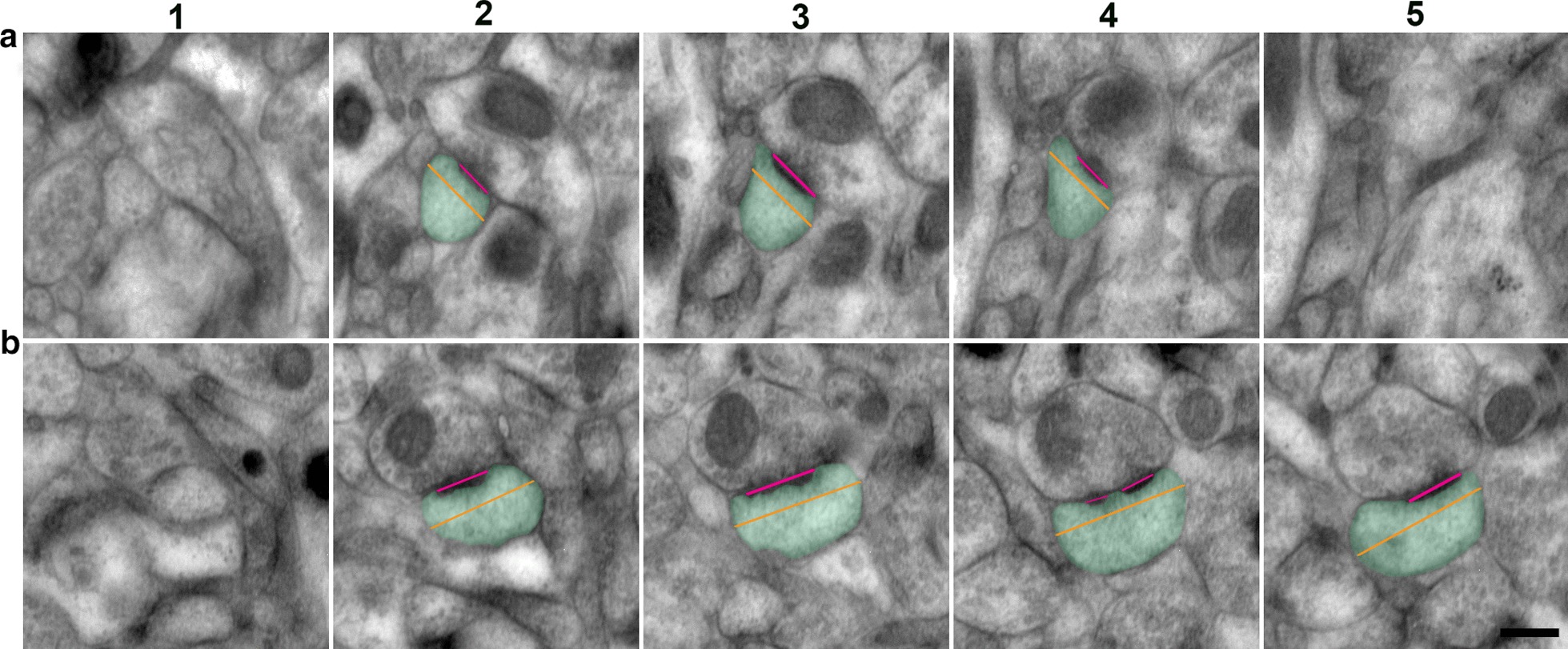
Measurement of PSD length and HD in non-perforated and perforated synapses. Electron micrographs were selected from two sets of five serial sections each to depict the synapse types. **a** A unique non-perforated synapse and **b** a perforated synapse. The sections are numbered 1–5 to show their position in the series. The postsynaptic spine of unique synapses is indicated in green. PSD length (magenta line) and HD (orange line) were measured for each non-perforated synapse and in all the sections where it was visible for the perforated synapse. Scale bar = 250 nm

Axospinous synapses were identified by their asymmetric PSD, a clear synaptic cleft, and the presence of vesicles in the presynaptic axonal bouton. Synapses were analyzed by the disector method [61], as described in our previous studies [57, 62]. Briefly, we identified synapses across two pairs of layers, the reference layer (sections one and five) and the lookup layer (sections two and four), from five serial sections and counted only the unique synapses found either in the reference layer or in the lookup layer (Fig. 1). Perforated synapses were identified by the presence of a clear gap in the middle of the PSD in any of the layers where the PSD was visible starting from the unique synapse in the second layer (Figs. 1, 2). We subtracted the number of perforated synapses from that of the total synapses to obtain the number of non-perforated synapses. The disector area was 42.77 μm^2^, and disector height was 0.18 μm; therefore, synapse density was calculated as the total number of counted synapses of each type (perforated, non-perforated, and total), from both the first and second pair of layers, divided by the total volume of the disector (7.70 μm^3^).

The length of the PSD and the maximal HD were measured starting with the unique PSDs identified in the first set of lookup layers (sections 2 and 4 in Fig. 2) and proceeding through the series either forward (from section 2 to 5 in Fig. 2) or in reverse (from from section 4 to 1 in Fig. 2). For non-perforated synapses, we measured the maximal length of the PSD (Fig. 2a). For perforated synapses, we added each length of the different segments of the PSD to obtain the maximal PSD length (Fig. 2b). The HD was measured using the maximal length of the spine head parallel to the PSD (Fig. 2). The PSD length and the HD were measured in all four layers if they were visible (Fig. 2). For the HD, the largest HD among all four layers was represented as the maximal HD of the synapse (Fig. 2). Similarly, for the PSD length, only the longest PSD length was used. However, the PSD area was calculated by multiplying the PSD length with the thickness of the layer (0.09 μm) and adding together the results for each layer. The measurements of PSD length and HD were taken at a resolution of 0.5 μm/137 pixels.

### Statistical analysis

Genotype effects on basal dendritic length were compared using one-way analysis of variance (ANOVA) followed by Bonferroni’s multiple comparison test. Effects of genotype and distance from the soma on Sholl analysis of dendritic length and intersections were compared using two-way ANOVA followed by Bonferroni’s post hoc test. Comparisons of potential genotypic variations in density and morphology of spines and synapses were determined using one-way ANOVA corrected for multiple comparisons using the Tukey’s post hoc test. Calculations were performed and graphs plotted using GraphPad Prism (GraphPad Software, San Diego, CA). Cumulative distributions of spine head diameter and volumes were plotted in MATLAB (R2020a, MathWorks, Natick, MA), compared using Kolmogorov–Smirnov test, and corrected for multiple comparisons using the Benjamini–Hochberg test. Statistical significance was set at an *α* level of 0.05, and data were shown as mean ± S.E.M.

## Results

### Dendritic morphology was comparable in *Shank3*-deficient rats and controls

**Fig. 3:**
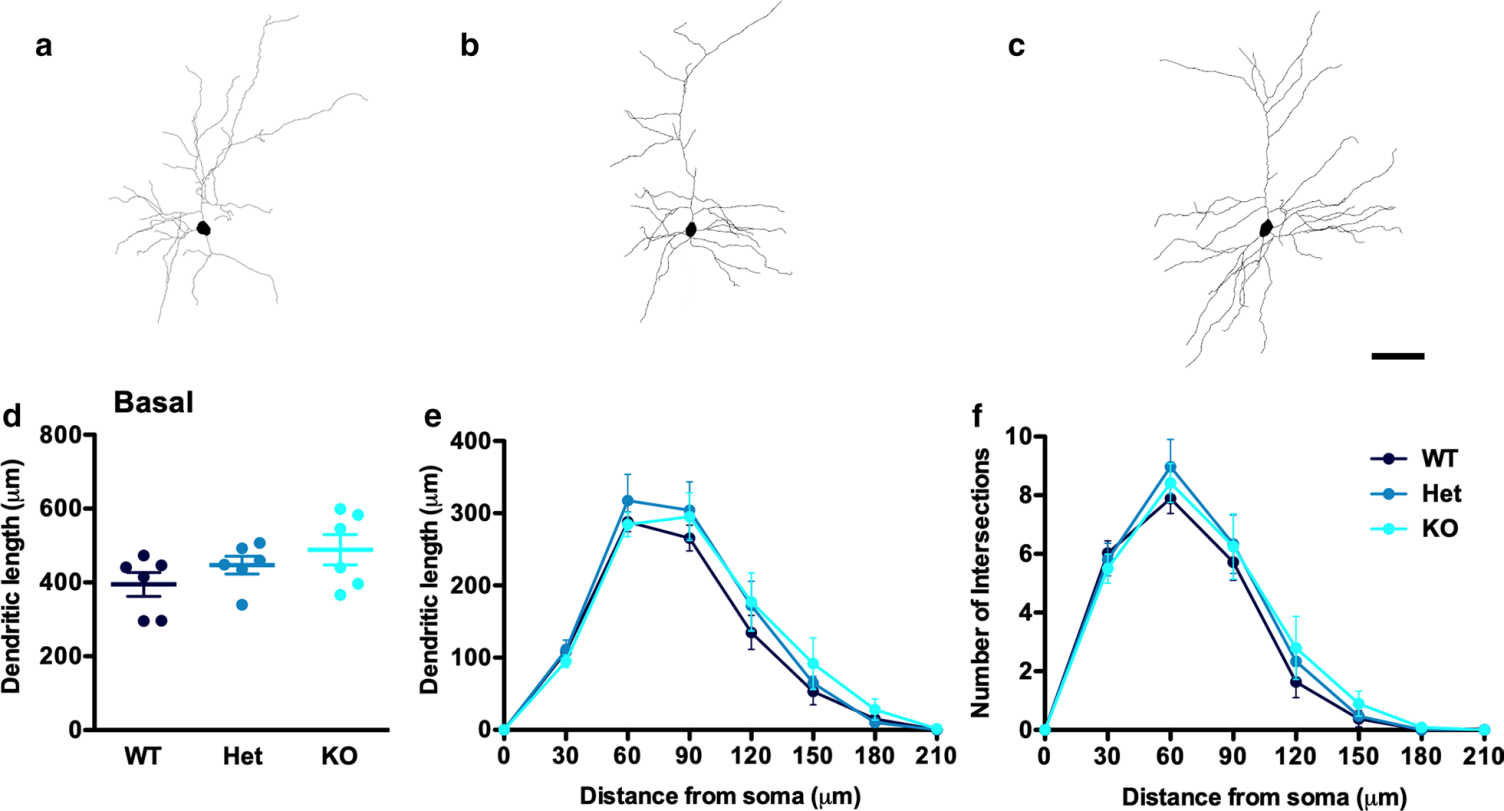
Basal dendritic length was comparable in WT, *Shank3*-Het, and *Shank3*-KO. Examples of dendritic reconstructions from Lucifer-Yellow filled layer II–III pyramidal neurons in the mPFC of **a** WT, **b** *Shank3*-Het, and **c** *Shank3*-KO rat. Scale bar = 50 µm. **d** Mean dendritic length was comparable among the three genotypes. Sholl analysis at 30-µm intervals from the soma revealed no changes in **e** dendritic length and **f** number of intersections among the three genotypes. WT, wild-type; Het, *Shank3* heterozygotes; KO, *Shank3* knockouts

Basal and apical dendrites were reconstructed from WT (Fig. 3a), *Shank3*-Het (Fig. 3b), and *Shank3*-KO rats (Fig. 3c). As the number of complete apical dendrites from each animal was insufficient for analysis, the data on arborization and spines of apical dendrites are not included here. Basal dendrites from 6 neurons were reconstructed from each animal (Table 1).

**Table 1:** Summary of number of animals, neurons, and spines in the confocal data by genotype

	WT	Het	KO
Animals	3 males, 3 females	3 males, 3 females	3 males, 3 females
Neurons	36 (6 each)	36 (6 each)	36 (6 each)
Total spines	10,022 (1511–2009)	9739 (1456–1793)	9633 (1340–1828)
Thin	7798 (1130–1631)	7247 (1035–1368)	7350 (973–1333)
Stubby	1280 (159–287)	1710 (169–365)	1511 (130–358)
Mushroom	707 (62–198)	495 (48–121)	547 (77–144)
Filopodia	38 (0–14)	94 (9–22)	75 (3–27)
Branched	199 (7–55)	193 (1–92)	150 (7–42)

Data show total counts for each category, with the numbers in parentheses indicating the range

The mean length of basal dendrites was comparable among the WT, *Shank3*-Het, and *Shank3*-KO (*F*_[2,15]_ = 2.02, *p* = 0.17; Fig. 3d). Furthermore, Sholl analysis at 30-µm incremental distance from the cell soma revealed no effect of genotype on dendritic length (*F*_[2,120]_ = 1.19, *p* = 0.31; Fig. 3e) or number of intersections (*F*_[2,105]_ = 0.23, *p* = 0.80; Fig. 3f) of basal dendrites.

### Basal spine density and morphology were unchanged in *Shank3*-deficient rats

**Fig. 4:**
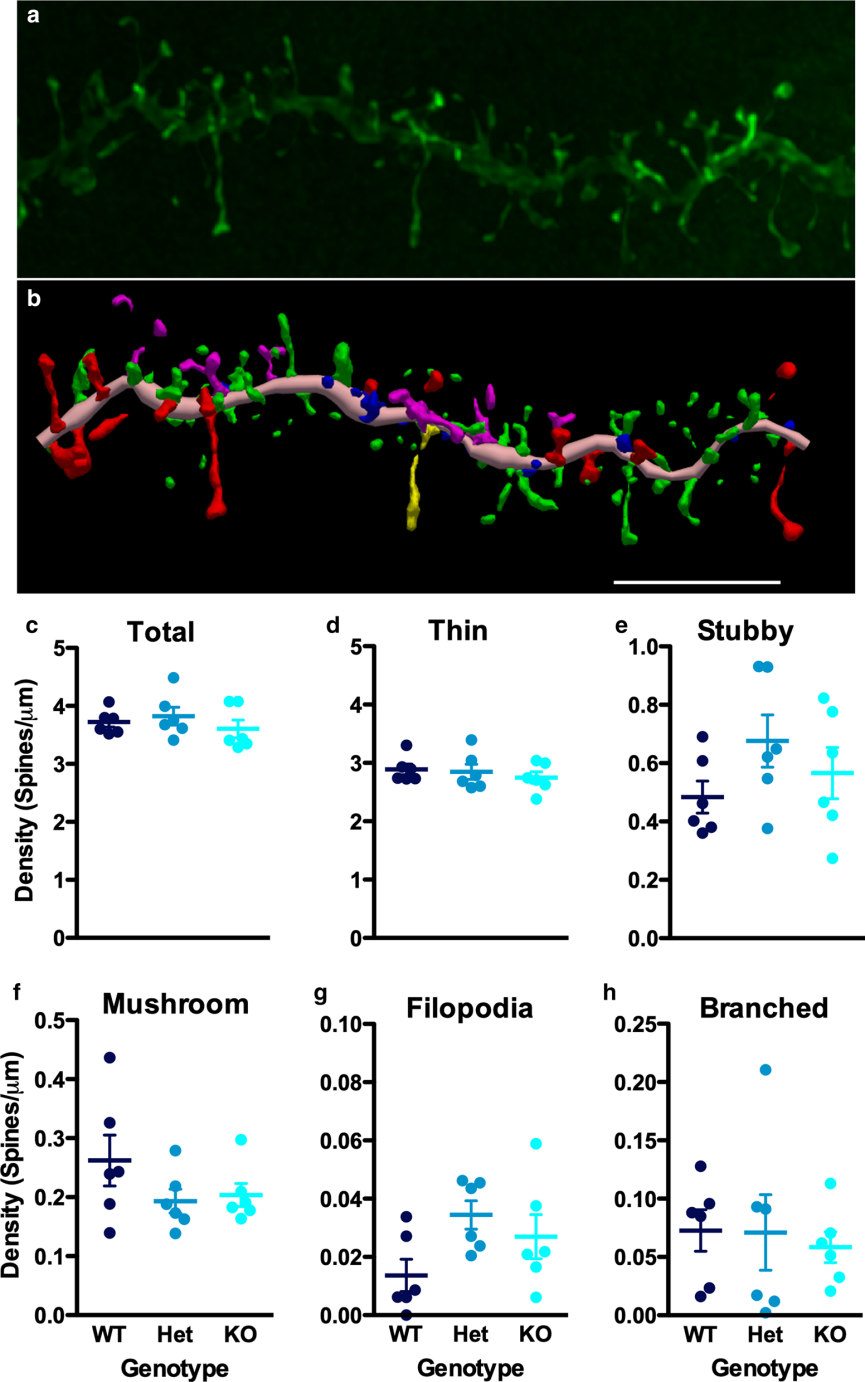
Spine density and morphology were similar in WT, *Shank3*-Het, and *Shank3*-KO. **a** Representative example of a deconvolved confocal image of a basal dendritic segment and **b** its 3D reconstruction, with the dendritic segment shown in light pink, thin spines in green, stubby in blue, mushroom in red, filopodia in yellow, and branched spines in magenta. Scale bar = 5 µm. **c** Total spine density and density by spine type **d** thin, **e** stubby, **f** mushroom, **g** filopodia, and **h** branched were comparable among the three genotypes. Note the difference in the scale of the Y-axes. WT, wild-type; Het, *Shank3* heterozygotes; KO, *Shank3* knockouts

Deconvolved confocal images of basal dendritic segments (Fig. 4a) were reconstructed in three dimensions (Fig. 4b) to analyze spine density and morphology (Table 1). There was no effect of genotype on total spine density (*F*_[2,15]_ = 0.67, *p* = 0.53; Fig. 4c). Spine densities analyzed by the different classes of spines, whether thin (*F*_[2, 15]_ = 0.44, *p* = 0.65; Fig. 4d), stubby (*F*_[2, 15]_ = 1.49, *p* = 0.26; Fig. 4e), mushroom (*F*_[2, 15]_ = 1.56, *p* = 0.24; Fig. 4f), filopodia (*F*_[2, 15]_ = 2.97, *p* = 0.08; Fig. 4g), or branched (*F*_[2, 15]_ = 0.12, *p* = 0.89; Fig. 4h), were also comparable among the three genotypes.

**Fig. 5:**
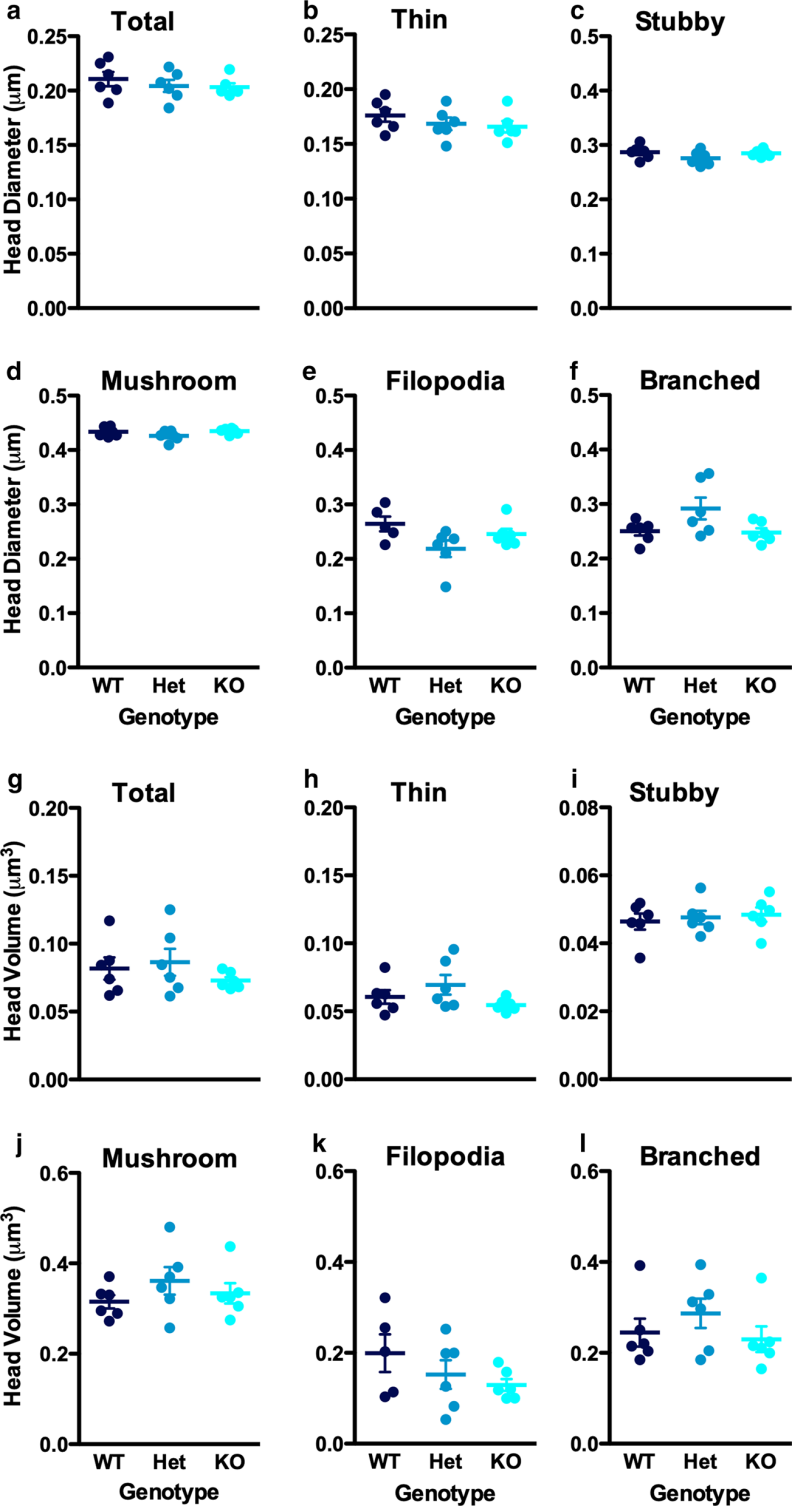
Spine HDs and head volumes were similar in WT, *Shank3*-Het, and *Shank3*-KO. HD of **a** total, **b** thin, **c** stubby, **d** mushroom, **e** filopodia, and **f** branched spines were comparable among the three genotypes. Head volumes of **g** total, **h** thin, **i** stubby, **j** mushroom, **k** filopodia, and **l** branched spines were similar among the three genotypes. Note the difference in the scales of the Y-axes. WT, wild-type; Het, *Shank3* heterozygotes; KO, *Shank3* knockouts

A comparison of spine HDs among the various spine classes revealed no significant effect of genotype on total (*F*_[2, 15]_ = 0.56, *p* = 0.58; Fig. 5a), thin (*F*_[2, 15]_ = 0.92, *p* = 0.42; Fig. 5b), stubby (*F*_[2, 15]_ = 1.74, *p* = 0.21; Fig. 5c), mushroom (*F*_[2, 15]_ = 2.07, *p* = 0.16; Fig. 5d), filopodia (*F*_[2,14]_ = 3.02, *p* = 0.08; Fig. 5e), or branched spines (*F*_[2, 15]_ = 3.5, *p* = 0.06; Fig. 5f). Spine head volumes were also comparable among the three genotypes for total (*F*_[2, 15]_ = 0.83, *p* = 0.46; Fig. 5g), thin (*F*_[2, 15]_ = 2.08, *p* = 0.16; Fig. 5h), stubby (*F*_[2, 15]_ = 0.23, *p* = 0.8; Fig. 5i), mushroom (*F*_[2, 15]_ = 0.98, *p* = 0.4; Fig. 5j), filopodia (*F*_[2,14]_ = 1.37, *p* = 0.29; Fig. 5k), and branched spines (*F*_[2, 15]_ = 0.94, *p* = 0.41; Fig. 5l).

**Fig. 6:**
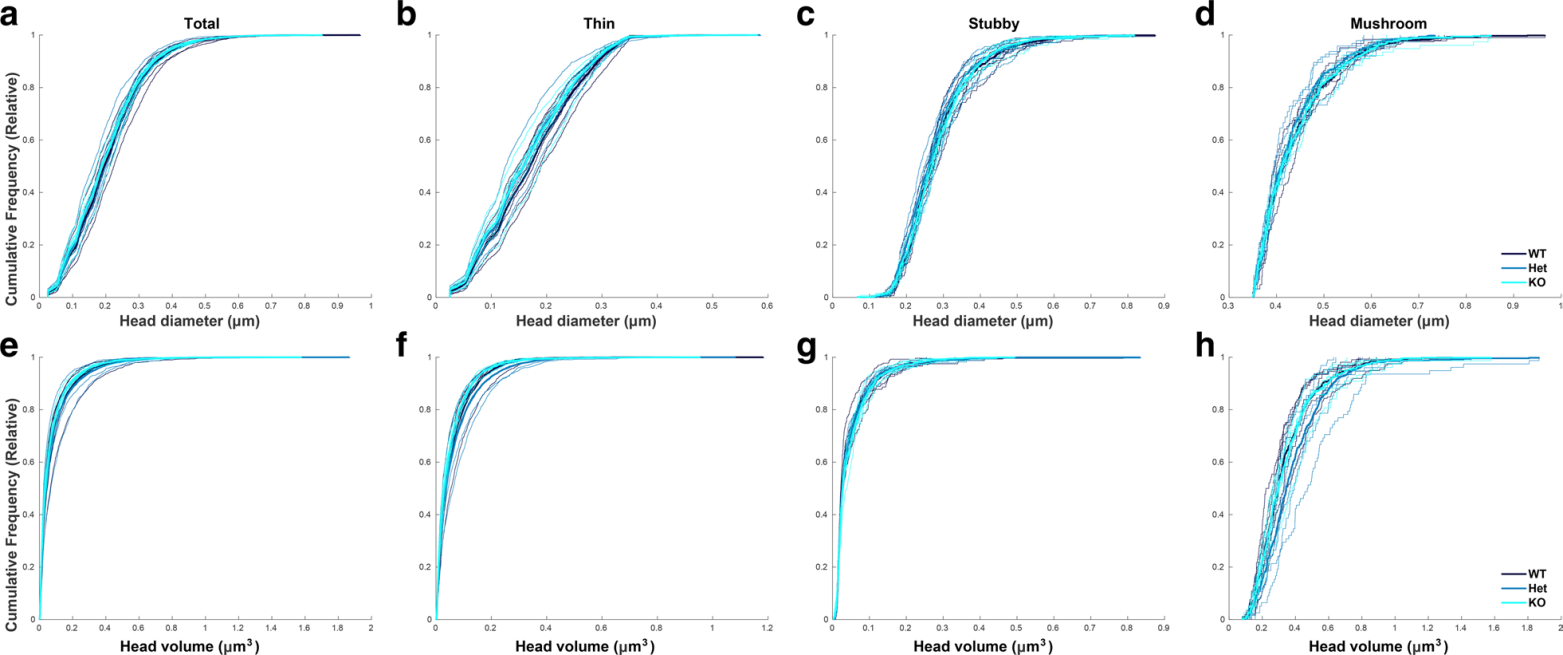
Cumulative frequency distributions of spine HDs and head volumes showed differences by spine class. HD of **a** total, **b** thin, and **c** stubby, but not **d** mushroom spines, and head volume of **e** total, **f** thin, **g** stubby, and **h** mushroom spines showed subtle differences among the three genotypes. Notably, the frequency of head volumes of thin and mushroom spines was shifted to the right in *Shank3*-Het compared to WT and *Shank3*-KO. WT, wild-type; Het, *Shank3* heterozygotes; KO, *Shank3* knockouts

Analysis of the cumulative frequency distribution of total spine HDs revealed that *Shank3*-Het and *Shank3*-KO rats were significantly different from the WT (*p* = 0.0008 and 0.0001, respectively; Fig. 6a). This difference was also evident in the HD of thin spines (both *p* < 0.0001; Fig. 6b). The distribution of stubby spines in *Shank3*-Het rats was significantly different than that in *Shank3*-KO (*p* = 0.0258; Fig. 6c). The distribution of mushroom (Fig. 6d), filopodia, and branched spine HD was comparable among the three genotypes.

Cumulative frequency distribution of total spine head volumes was significantly different in *Shank3*-KO rats compared to *Shank3*-Het and WT (both *p* < 0.0001; Fig. 6e). In the distribution of thin spines, *Shank3*-Het and *Shank3*-KO rats were significantly different from WT and from each other (all *p* < 0.0001, Fig. 6f). The distribution of stubby spines in *Shank3*-KO rats was significantly different from WT (*p* = 0.0205; Fig. 6g). Notably, the distribution of spine head volumes was shifted to the right in *Shank3*-Het compared to the other two genotypes for both thin (both *p* < 0.0001, Fig. 6f) and mushroom spines (both *p* < 0.0001, Fig. 6h), indicating more frequent large head volumes. There was no significant difference in the distribution of head volumes among the three genotypes for filopodia and branched spines.

### Synapse density was unchanged in *Shank3*-deficient rats compared to controls

For quantifying synapse density, approximately 7000 unique synapses (mean = 389 and range = 287–480 per animal; Table 2) were counted, with an average of 351 non-perforated and 38 perforated synapses per animal (Table 2).

**Table 2:** Summary of number of synapses included in EM data by genotype

	WT	Het	KO
Animals	6 males	6 males	6 males
Synapse density			
Total	2150 (287–434)	2523 (387–448)	2336 (338–380)
Non-perforated	1947 (271–380)	2276 (348–406)	2099 (306–431)
Perforated	203 (16–54)	247 (28–55)	237 (26–51)
Reconstructed synapses			
Total	1034 (155–194)	1213 (175–214)	1130 (156–253)
Non-perforated	835 (128–153)	972 (137–178)	899 (127–202)
Perforated	199 (17–55)	241 (31–53)	231 (26–51)

Data show total counts for each category, with the numbers in parentheses indicating the range

**Fig. 7:**
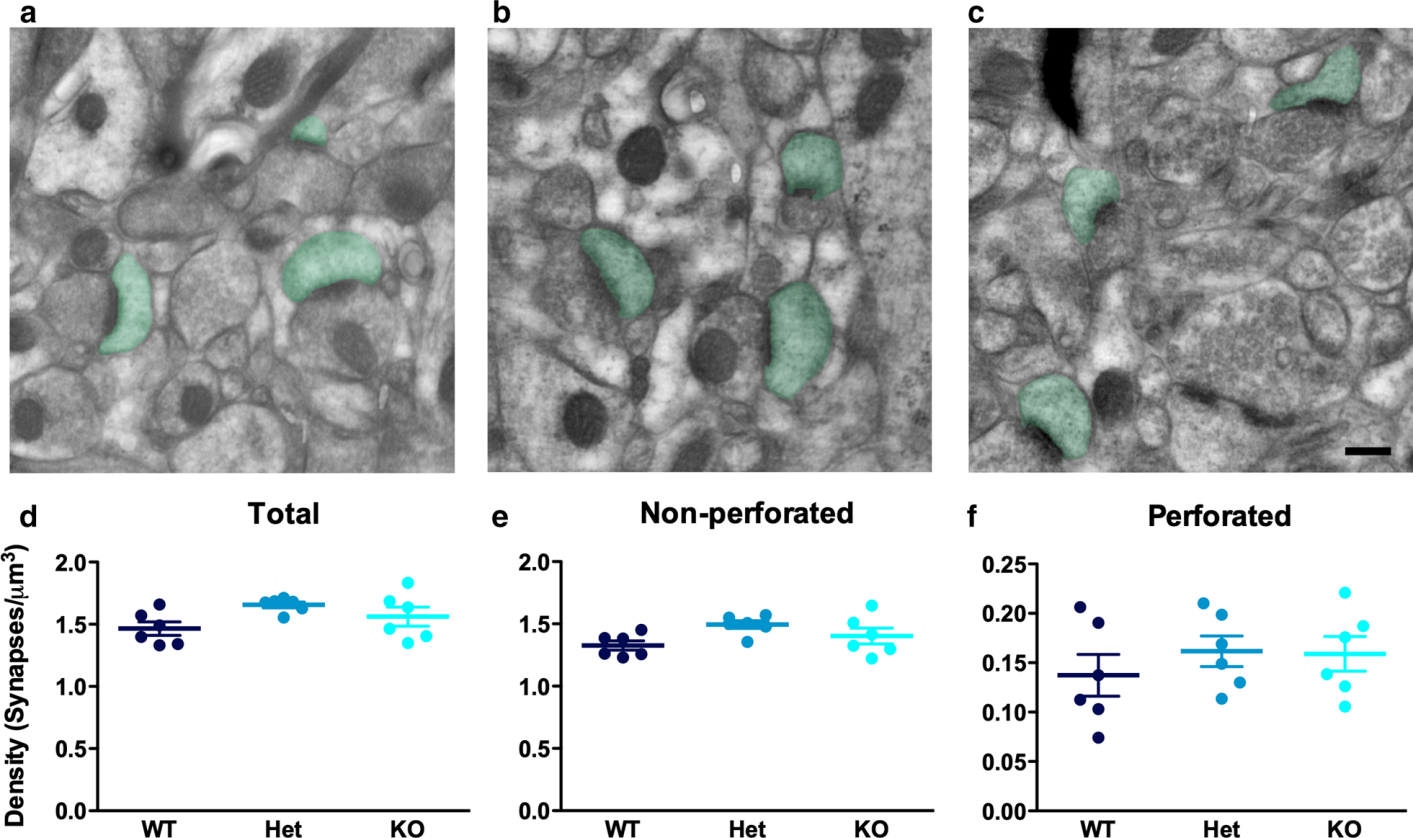
Synapse densities were comparable in WT, *Shank3-*Het, and *Shank3-*KO rats. Representative sections from **a** WT, **b** *Shank3-*Het, and **c** *Shank3-*KO rats showing unique synapses. The postsynaptic spine of unique synapses is indicated in green. Scale bar = 250 nm. **d** Total synapse density, **e** non-perforated synapse density, and **f** perforated synapse density were similar in all three groups. WT, wild-type; Het, *Shank3* heterozygotes; KO, *Shank3* knockouts

We did not find significant differences in total synapse density among *Shank3*-Het, *Shank3*-KO, or WT control rats (*F*_[2, 15]_ = 2.95, *p* = 0.08, Fig. 7a–d). Non-perforated (Fig. 7e) and perforated synapse densities (Fig. 7f) were also comparable (*F*_[2, 15]_ = 3.29, *p* = 0.07 and *F*_[2, 15]_ = 0.54, *p* = 0.60, respectively) among the three genotypes.

### Subtle changes in synaptic ultrastructure were present in *Shank3* heterozygotes

**Fig. 8:**
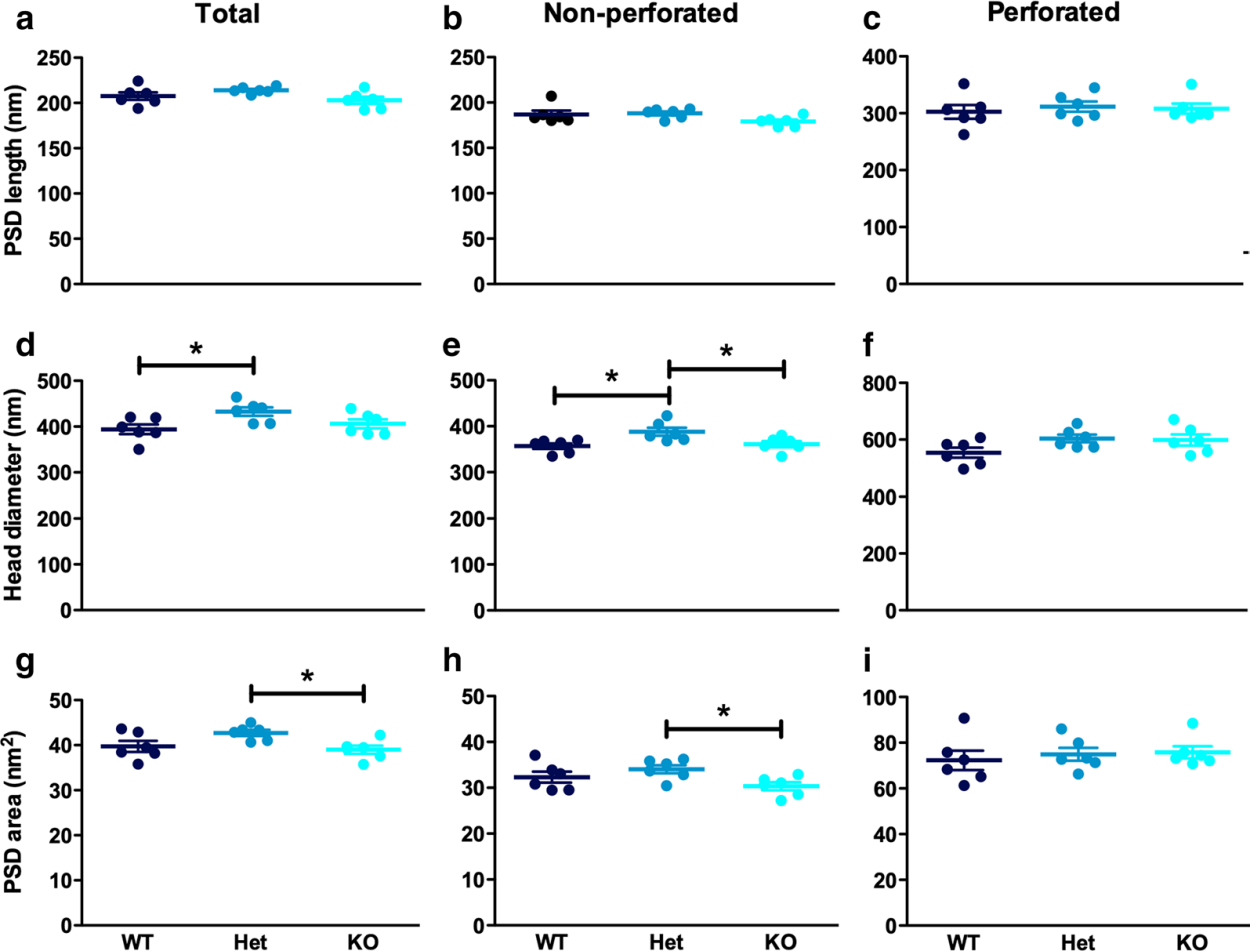
PSD length was comparable, but HD and PSD area were higher in *Shank3-*Het. PSD length in **a** total synapses, **b** non-perforated synapses, and **c** perforated synapses are comparable among WT, *Shank3*-Het, and *Shank3*-KO rats. **d** HD in total synapses is wider in *Shank3*-Het rats compared to the WT. **e** HD in non-perforated synapses is wider in *Shank3*-Het rats compared to both WT and *Shank3*-KO, but non-perforated synapses in the *Shank3*-KO have similar HD as the WT. **f** HD of perforated synapses is comparable in WT, *Shank3*-Het, and *Shank3-*KO. The *Shank3*-Het group has greater PSD area of **g** total and **h** non-perforated synapses compared to the *Shank3*-KO, but the *Shank3*-KO and WT are comparable for these measures. **i** PSD area of perforated synapses is similar among the three groups. WT, wild-type; Het, *Shank3* heterozygotes; KO, *Shank3* knockouts. *p < 0.05 by one-way ANOVA followed by Tukey’s test compared to the indicated group

In order to measure PSD length, PSD area, and HD, approximately 150–250 synapses were reconstructed per animal, including 128–202 non-perforated and 17–55 perforated synapses (Table 2). There were no significant differences in total PSD length when comparing *Shank3*-Het, *Shank3*-KO, and WT rats (*F*_[2, 15]_ = 2.74, *p* = 0.10; Fig. 8a). Additionally, the PSD lengths of non-perforated (*F*_[2, 15]_ = 2.73, *p* = 0.10, Fig. 8b) and perforated synapses (*F*_[2, 15]_ = 0.21, *p* = 0.81; Fig. 8c) were comparable between *Shank3*-deficient and WT rats.

However, maximal HD was significantly altered in the *Shank3*-Het group, but not in the *Shank3*-KO, when compared to the WT. Total HD was significantly higher (*F*_[2, 15]_ = 4.08, *p* = 0.04) in the *Shank3*-Het rats compared to the WT (Fig. 8d). We also found wider (*F*_[2, 15]_ = 5.67, *p* = 0.01) HD of non-perforated synapses in the *Shank3*-Het group compared to both WT and *Shank3*-KO rats (Fig. 8e). When we analyzed only the perforated synapses, there was no significant change in HD among the groups (*F*_[2, 15]_ = 2.60, *p* = 0.11; Fig. 8f).

The total PSD area was greater (*F*_[2, 15]_ = 4.28, *p* = 0.03) in the *Shank3*-Het rats compared to *Shank3*-KO (Fig. 8g). This change was evident in non-perforated synapses (*F*_[2, 15]_ = 3.38 and *p* = 0.06 when comparing all three groups, *p* < 0.05 between *Shank3*-Het and KO; Fig. 8h), as there was no significant change in PSD area of perforated synapses among the three groups (*F*_[2, 15]_ = 0.30, *p* = 0.75; Fig. 8i).

## Discussion

The interaction of Shank proteins with their various partners is regulated by several mechanisms. First, alternative exon splicing results in multiple splice variants of Shank3 in the human brain [17]. Splice sites are present downstream of the SH3 and PDZ domains and within the PRC and SAM domains of Shank3 [25], and the human brain shows higher expression of exons encoding these functional regions [63]. Second, Shank3 has six intragenic promoters, resulting in differential expression of isoforms, named Shank3a through Shank3f, during brain development [25, 64, 65]. Finally, the subcellular and tissue-specific patterns of expression and turnover of Shank isoforms during development and synaptic activity are also regulated by methylation [66–68] and ubiquitination [69].

The Shank3 rat model used here demonstrated attentional deficit and reduced hippocampus-to-mPFC signaling in both *Shank3*-Het and *Shank3*-KO rats compared to WT [55], pointing toward a synaptic deficit in the mPFC. The synaptic deficits may be due to changes in dendrite or spine morphology, altered synapse density, or decreased recruitment of proteins to the PSD that would be visible as ultrastructural changes of the PSD or spine head. We found no significant changes in complexity of basal dendrites in layer III pyramidal neurons of the mPFC of *Shank3*-Het and *Shank3*-KO rats compared to WT. Similar results were previously reported in the hippocampus of *Shank3*-KO mice with an exon 21 deletion [70]. Other studies showed increased dendritic length and complexity in striatal medium spiny neurons of *Shank3*-KO mice with a deletion of exons 13–16 [71] and increased complexity in Purkinje neurons in the cerebellum of *Shank3*-Het mice with an exon 21 deletion [72], suggesting a brain-region- or cell type-specific effect of decreased Shank3 expression on dendritic arborization. Our results show no significant change in the total density of excitatory synapses among the three experimental groups at 5 weeks of age. Although few studies have assessed synapse density per se, our results are corroborated both by our current finding of no changes in spine density and by previous reports showing no change in spine density in the neocortex of *Shank3*-Het mice [73] and in the hippocampus of *Shank3*-KO mice with an exon 21 deletion [70]. However, other studies have reported a significant decrease in spine density in *Shank3*-deficient models compared to controls, as observed in neurons differentiated from human-induced pluripotent stem cells carrying *SHANK3* mutations [74], the PFC of a macaque model with deletions in exons 6 and 12 [75], the hippocampus of 5-week-old *Shank3*-KO rats with deletion of exons 11–21 and loss of all the Shank3 isoforms [76], the striatum [71] and the anterior cingulate cortex [77] of *Shank3*-KO mice lacking Shank3b following deletion of exons 13–16 containing the PDZ domain, and the cerebellum of *Shank3*-Het mice with an exon 21 deletion [72]. However, *Shank3*-KO mice with an exon 4–9 deletion and loss of the Shank3a and Shank3b isoforms bearing the ANK and SH3 domains showed decreased spine density in the hippocampus at 4 weeks of age, but no change at 10 weeks [78]. *Shank3*-KO mice with exons 4–22 deleted and a complete loss of all Shank3 isoforms and splice variants showed decreased spine density only in the striatum, not the hippocampus, at 8 weeks of age [79]. In vitro studies have shown that full-length Shank3 was localized in dendritic spines, whereas a C-terminal truncated isoform was diffusely distributed in dendrites and axons [64] and the isoform length determined its effects on dendritic spine density [65]. Thus, the age of the animal, the extent of *Shank3* deletion, and the brain regions examined all determined whether synaptic density was altered in Shank3-deficient animals.

Dendritic spines are classified as thin, stubby, or mushroom [80] based on their length and the relative size of their head and neck, or branched when several heads originate from a single neck on the dendrite [81]. The long, thin filopodia are thought to be precursors in the formation of spines [82]. Spines and filopodia are highly dynamic during developmental stages and in response to stimulation [83]. We did not observe any differences in the mean spine density of the various spine classes of *Shank3*-deficient rats compared to controls. Others have reported reduced density of mushroom spines in the anterior cingulate cortex [77] and increased thin spines in *Shank3*-KO mice lacking Shank3b due to deletion of exons 13–16 [84]. Although mean HD and head volumes were comparable among the three genotypes in our study, the shift in distribution toward larger head volumes for thin and mushroom spines on mPFC pyramidal neurons suggests subtle ultrastructural changes due to the loss of Shank3 as a synaptic effector in glutamatergic neurons in a spine-type specific manner. Stable enlargement of the more plastic thin spines and transient enlargement of mushroom spines [85], the latter implicated in long-term memory storage, could reflect morphologically the impaired behavioral phenotypes observed in this rat model of *Shank3* deficiency [55].

Synapses show different structures, with perforated or non-perforated PSDs, according to changing neurotransmission efficacy in response to activity [86, 87] and function [86–89]. We did not observe any differences in density of non-perforated or perforated synapses in the PFC among the three genotypes of rats. However, in the hippocampus of 5-week-old *Shank3*-Het mice where the ANK domain was deleted, non-perforated synapse density was unchanged but perforated synapse density was higher compared to the *Shank3*-KO and WT groups; this change was not present at 3 months of age [62]. The observed difference in density of the perforated synapses between the two Shank3 models may be due to different effects of Shank3 in the two brain areas studied.

Maximal PSD length was not significantly changed, but HD was increased in the *Shank3*-Het group and not in the *Shank3*-KO compared to WT rats. PSD area was higher in the *Shank3*-Het group compared to the *Shank3*-KO. The PSD area was calculated using the PSD length across the series of sections where the synapse was visible. The observed change in size of the spine head could also modify the PSD area. Notably, the change in HD was only detected at the nanometer-scale resolution provided by EM, not by our confocal analysis of spine HD or volume, indicating a subtle pathological change in morphology rather than altered neuronal integrity at the cellular level. Similar to our results, PSD length and thickness were unchanged in hippocampal CA1 neurons of *Shank3*-KO mice with an exon 4–9 deletion [78]. In a different model with a similar deletion, PSD length and area as well as HD were unchanged in the hippocampus of both *Shank3*-Het and *Shank3*-KO mice compared to WT at 5 weeks and 3 months of age [62]. These results were confirmed in the hippocampus of *Shank3*-KO mice with exon 4–21 deletion, but in the striatum of these mice, decreased PSD length and thickness were seen at 8 weeks of age [79]. Shorter and thinner PSDs were also reported in the anterior cingulate cortex of *Shank3*-KO with a deletion of exons 13–16 [77]. Of note, the increase in total PSD area and HD that we observed was present only in the *Shank3*-Het rats compared to either KO or WT and no change in the synaptic ultrastructure was observed in the *Shank3*-KO group compared to the WT. The *Shank3*-Het rats carry one copy of undeleted *Shank3* that can express the full-length isoforms of the protein, though at lower levels than the WT. *Shank3*-KO rats express no full-length Shank3 and the truncated isoforms in these animals may not be sufficient to recruit the Shank3-binding partners in the PSD that potentially compensate for this lack in the *Shank3*-Het rats. The *Shank3*-KO rats may be able to maintain the structure of dendritic spines and the PSD comparable to WT through the shorter Shank3 isoforms or by recruiting the other Shanks to the PSD. Although the ANK domain of Shank3 is deleted in our model, the synapse-targeting SAM domain as well as the major binding sites on Shank3 for recruiting the NMDA and AMPA receptors to the PSD and for binding cytoskeletal proteins essential for spine morphology are preserved in the *Shank3*-KO. These domains may be sufficient to maintain synaptic morphology at a level similar to that in the WT.

In the *Shank3*-Het rats that carry only one copy of the gene and can express full-length Shank3 together with the shorter isoforms, the other Shank family proteins may compensate for the loss of Shank3, resulting in the observed increase in size of the dendritic head and PSD area. Shank2, which shares both a homologous PRC domain and a synapse-targeting C-terminal region with Shank3 [22, 28], seems to be the better candidate to compensate for reduced Shank3 expression. Indeed, Shank2 with an intact PRC domain can rescue reduced head diameter of dendritic spines in hippocampal neurons, induced by a knockdown of all three Shank proteins [90]. Overexpression of Shank1 containing the PRC domain also results in enlargement of spine head size [91]. In contrast to our results, no change in head diameter and decreased spine head volume were observed in human neurons differentiated from induced pluripotent stem cells sourced from subjects with Shank3 mutations [74]. Furthermore, a deletion of the ANK-SH3 domains of Shank3 reduced spine head area in mouse hippocampal neurons [53]. In our *Shank3*-Het rat model, not only are low levels of the full-length Shank3 protein expressed, but also the shorter isoforms lacking the ANK domain but containing the PDZ domain which recruits NMDA and AMPA receptors during spine maturation [53], the PRC domain where Homer1 and cortactin bind [20, 21, 25], and the synapse-targeting SAM domain [64]. Thus, overcompensation by the full-length protein recruiting the shorter Shank3 isoforms or other Shank proteins and giving an enlargement of the HD and PSD area seems probable.

PSD fractions from the neocortex of *Shank3*-Het and *Shank3*-KO rats with an exon 11–21 deletion [76] and hippocampal neurons after knockdown of four major isoforms of Shank3 in vitro show no change in Shank1 or 2 expression [92], but other compensatory mechanisms may occur in vivo in the PFC. A study in the mouse brain found that proteins interacting with Shank3 in the PFC are different from those in the hippocampus and striatum [93]. PFC-specific epigenetic modifications of histones, such as higher dimethylation [94] and lower acetylation [95] in *Shank3*-Het mice with an exon 21 deletion and higher dimethylation in postmortem ASD brains [94], suggest a brain region-specific mechanism for regulation of protein expression. Notably, the expression of Shank3 is highest in the PFC compared to other regions in the macaque brain [75], whereas Shank3 isoforms containing ANK domain are highly expressed in the upper cortical layers, hippocampus, and striatum of the mouse brain [96]. Multimer formation of Shank proteins may enable their recruitment to the synapse without an overall increase in protein concentration. Shank3 forms multimers via its PRC and SAM domains [20], although these interactions remain to be proven endogenously in neurons. Interestingly, the interaction of ANK repeats and SH3 domains in Shank1 is reported to require all the repeats in the ANK domain [97] and, if this extends to Shank3, the lack of the ANK domain in Shank3 of the *Shank3*-KO rats could explain their difference from the *Shank3*-Het rats in our study. Despite these potential compensatory mechanisms for some functions when Shank3 is deficient, the presence of clinical phenotypes due to Shank3 haploinsufficiency in humans carrying *SHANK3* mutations [6, 7, 9, 13] and of behavioral deficits in animal models with Shank3 deficiency [55, 73, 76, 98] suggests such compensation may not be fully effective to rescue the functional phenotype but may be able to partially preserve the ultrastructure of synapses.

## Limitations

Although the prevalence of PMS is equal in both sexes [99], ASD occurs four times more frequently in males than in females [1]. The ultrastructural study used only male mice to uncover synaptic changes in the mPFC relevant to both ASD and PMS. The deletion in exon 6 of the Shank3 gene would affect the N-terminal ANK domain, truncating the full-length protein, but shorter isoforms of Shank3 may still be expressed in the *Shank3*-Het and *Shank3*-KO rats. Thus, the changes observed using this rat model would more accurately reflect the effects of mutations affecting the N-terminal of the protein than those resulting from deletion of the gene and the loss of all isoforms of the protein.

## Conclusions

The increase in PSD area and size of the spine head in the *Shank3*-Het rats, but not the *Shank3*-KO, may more accurately model synaptic changes resulting from haploinsufficiency of *SHANK3* that is linked to the neurological symptoms in PMS. These changes in the mPFC at the level of synaptic ultrastructure may have implications for the attentional deficit observed in *Shank3*-Het rats and also in subjects with PMS and ASD who carry mutations in *SHANK3*.

## Data Availability

The datasets analyzed during this study are available from the corresponding author on reasonable request.

## Abbreviations

PMS: Phelan–McDermid syndrome
ASD: Autism spectrum disorder
SHANK3: SH3- and Ankyrin-binding protein 3
mPFC: Medial prefrontal cortex
KO: Knockout
Het: Heterozygote
WT: Wild-type
PSD: Postsynaptic density
HD: Head diameter
PFA: Paraformaldehyde
PB: Phosphate buffer
ANOVA: Analysis of variance
GKAP: Guanylate kinase-associated protein
mGluR: Metabotropic glutamate receptor
ANK: Ankyrin repeats domain
SH3: SRC homology domain 3
PDZ: PSD-95/Disc Large homolog-1/Zonula occludens-1 domain
PRC: Proline-rich cluster domain
SAM: Sterile alpha-motif domain
NMDA: *N*-Methyl-d-aspartate
AMPA: Amino-3-hydroxy-5-methylisoxazole-4-propionic acid
